# A Primary Care Program Based on Behavioral Reeducation and Abdominal Massage for Improving the Symptoms of Chronic Constipation: Protocol for a Randomized Controlled Trial

**DOI:** 10.2196/72018

**Published:** 2025-12-04

**Authors:** Cristina Segura-Bayona, Josep Vidal-Alaball, Anna Ramírez-Morros, Queralt Miró-Catalina, Anna Ruiz-Comellas

**Affiliations:** 1 Primary Care Centre Igualada Urbà, Gerència d’Atenció Primària i Comunitària Penedès, Institut Català de la Salut, Passeig Jacint Verdaguer 170, Barcelona, 08700, Spain, +34 938053500; 2 Health Promotion in Rural Areas Research Group, Gerència d’Atenció Primària i a la Comunitat de la Catalunya Central, Institut Català de la Salut, Barcelona, Spain; 3 Central Catalonia Research Support Unit, Jordi Gol i Gurina University Institute for Research in Primary Health Care Foundation, Manresa, Spain; 4 Faculty of Medicine, Vic-Central University of Catalonia, Vic, Spain; 5 Intelligence for Primary Care Research Group, Jordi Gol i Gurina University Institute for Research in Primary Health Care Foundation, Manresa, Spain; 6 Primary Care Centre Sant Joan de Vilatorrada, Gerència d’Atenció Primària i a la Comunitat de la Catalunya Central, Institut Català de la Salut, Sant Joan de Vilatorrada, Spain

**Keywords:** primary health care, proctologic disease, pelvic floor disorders, physical therapy, health education

## Abstract

**Background:**

Chronic constipation is a prevalent and often underestimated gastrointestinal disorder that significantly affects quality of life, particularly among women and older adults. In Spain, it is estimated to affect between 12% and 20% of the population, contributing to increased health care visits, economic costs, and medication dependency. Although pharmacological treatments such as laxatives are widely used, they often offer only temporary relief and may lead to adverse effects or dependency. There is growing interest in nonpharmacological interventions that address the root behavioral and functional causes of constipation, such as dietary habits, physical inactivity, and impaired defecation techniques. However, evidence regarding the effectiveness of such approaches, especially within a primary care setting, remains limited.

**Objective:**

This study aims to evaluate the effectiveness of a primary care–based structured rehabilitation program that combines behavioral reeducation and abdominal massage therapy in reducing the severity of chronic constipation and use of laxatives. A secondary aim is to assess improvements in quality of life and sustainability of effects over time.

**Methods:**

This is a randomized controlled trial involving adults aged 18 to 75 years from the counties of l’Anoia and Bages (Catalonia, Spain) who meet the Rome IV diagnostic criteria for chronic constipation. A total of 45 participants will be randomly assigned in a 1:1:1 ratio (approximately 22‐23 per center, with 15 per group overall) into 3 groups: a control group, behavioral intervention (BI) group, and behavioral intervention and massage (BIM) group. All participants will complete baseline assessments that include the Rome IV criteria, the Bristol Stool Form Scale, the CVE-20 quality of life questionnaire, and the International Physical Activity Questionnaire. The BI group will receive 2 group education sessions, focused on healthy bowel habits, diet, hydration, physical activity, stress management, and medication use, delivered by a multidisciplinary team. The BIM group will receive the same intervention as the BI group, plus two 30-minute sessions with a physiotherapist to learn abdominal self-massage techniques. The control group will receive usual care. Follow-up assessments will occur at 3 and 6 months after the intervention using the same instruments and a self-recorded calendar of laxative use and massage application. Data will be analyzed using appropriate statistical tests, including the *χ*^2^ test, a 2-tailed *t* test, and ANOVA/Kruskal-Wallis tests, depending on variable type.

**Results:**

Participant enrollment concluded in August 2025, and data collection is ongoing and expected to continue until April 2026.

**Conclusions:**

This trial will provide evidence on the efficacy of conservative, low-risk interventions for managing chronic constipation in primary care. The findings may support broader implementation of integrative approaches that reduce pharmacological dependence and enhance patient empowerment, with potential public health and economic benefits.

## Introduction

Constipation is characterized by difficult or infrequent passage of stool, often accompanied by straining, pain, or a feeling of incomplete evacuation. It is one of the most common digestive problems in today’s society and contributes significantly to decreased quality of life, increased medical visits, and high costs to the health care system [1].

Constipation may be acute, usually lasting less than 6 weeks, or chronic, lasting more than 3 months. Most often, chronic constipation is the result of a primary disorder, that is, one caused by dietary factors (such as insufficient fiber intake), lifestyle factors (lack of mobility or a sedentary lifestyle), or a disorder causing deficient colonic propulsion or rectal emptying [2].

The prevalence of constipation differs across countries due to varying lifestyles and diet. On a global scale, it is between 10% and 15% [3]. According to the American Gastroenterological Association, the prevalence in the United States is approximately 16% in adults and 33% in people older than 60 years [4]. In Europe, it varies between 8% and 26% [5]. In East Asia, it has been reported that in China the prevalence of this pathology is 8.5% [6]. In the African pediatric population, it is 31.4% [7], with no data for the adult population. There is a lack of prevalence studies in South America, West Asia, and Africa.

The Fundación Española del Aparato Digestivo (Spanish Foundation of the Digestive System) estimates that constipation affects between 12% and 20% of the Spanish population [8]. On the other hand, Navarro-Ruiz et al [9], in a study conducted in the primary care setting in 2015, stated that it varied between 16% and 33% of the population.

An analysis of prevalence based on factors such as gender, age, physical activity level, and medication use in the population shows that constipation tends to be more frequent in women (at a 2:1 ratio), older people, people with low physical activity levels, and people taking medications with digestive side effects (eg, opioids, anticholinergics, and calcium channel blockers) [2-5,8-10]. Therefore, this condition is expected to increase in the coming years due to the prevalence of sedentary lifestyles and aging populations, especially in developed countries.

The diagnostic criteria for chronic constipation are the Rome IV criteria, shown in Textbox 1 [11]. Criteria must have been met for at least the last 3 months and symptoms must have started at least 6 months prior to diagnosis.

Textbox 1. **Rome IV criteria for constipation.**

1. Two or more of the following criteria are met:
  - Excessive straining in at least 25% of bowel movements
  - Hard stools in at least 25% of the stools (Bristol type 1-2)
  - Sensation of incomplete evacuation in at least 25% of bowel movements
  - Sensation of anorectal obstruction or blockage in at least 25% of bowel movements
  - Less than 3 complete spontaneous bowel movements per week
2. The presence of liquid stool is rare without the use of laxatives.
3. Criteria are not met for the diagnosis of irritable bowel syndrome.

*Acute* constipation rarely causes complications or long-term health problems. Treatment is usually effective, especially if started promptly. However, long-term, chronic constipation is likely to pose a higher risk of complications [12] such as fecal incontinence [13], hemorrhoids (4%) [14], anal fissure (7.8%) [14], organ prolapse [15], fecal impaction [16] or stercoral perforation [17,18].

The current treatments available for this condition are limited: lifestyle modification, medication, and surgical intervention when the condition is severe [19-23]. Laxative treatment is effective, but there are often relapses after withdrawal, causing dependence in some patients. In addition, there are side effects such as electrolyte disorders, dehydration, and intestinal cramps [24]. Therefore, this condition calls for new multidisciplinary and conservative strategies, especially within primary care, that are accessible, low-risk, and empower patients in self-care.

There is growing evidence that abdominal massage can stimulate colonic motility, reduce transit time, and promote defecation through mechanical and neurophysiological mechanisms involving the enteric nervous system and visceral reflexes [25,26]. Behavioral reeducation, including guidance on bowel habits, posture, and toileting routines, has also been shown to improve constipation outcomes by targeting dysfunctional defecation behaviors and increasing patient awareness and adherence to lifestyle recommendations [27]. These approaches align with biopsychosocial models of gastrointestinal dysfunction, emphasizing nonpharmacological, patient-empowering strategies.

The primary objective of this study is to evaluate the effectiveness of a structured rehabilitation program based on behavioral reeducation and abdominal massage therapy in reducing the prevalence of chronic constipation and the use of laxatives at 3, 6, and 12 months after the intervention. The secondary objectives are to assess changes in quality of life among participants and to evaluate whether the observed effects are sustained over time.

## Methods

This protocol has been developed and drafted in accordance with the recommendations of the SPIRIT (Standard Protocol Items: Recommendations for Interventional Trials) guide [28].

### Design: Randomized Controlled Trial

This will be a randomized controlled trial with 3 groups: a control group, a behavioral intervention (BI) group, and a behavioral intervention and massage (BIM) group. The same number of people will be randomly distributed in each group with a 1:1:1 allocation ratio, so that the 3 groups are homogeneous for age, sex, and constipation characteristics. Data collection will be between 2024 and 2026.

### Scope: Primary Care

The choice of primary care as the study setting reflects its central role in the early management and long-term monitoring of chronic constipation. This environment ensures high feasibility, as the intervention can be delivered using existing staff—nurses, physiotherapists, general practitioners, and nutritionists—and infrastructure, requiring minimal additional resources. Moreover, the intervention is designed to be easily integrated into routine care pathways, aligning with preventive care goals and enhancing patient self-management strategies within the primary care framework.

### Population

This study will recruit patients aged 18 to 75 years assigned to the primary care centers of the counties of l’Anoia and Bages in Catalonia, Spain.

The inclusion criteria are having been diagnosed with primary constipation using the Rome IV criteria [11] lasting for more than 3 months and understanding Catalan, Spanish, or English.

The exclusion criteria are having constipation secondary to neurogenic, metabolic, endocrine, or postoperative diseases; having constipation secondary to medication for other pathologies, the medication list for which can be found in Multimedia Appendix 1; having any type of open abdominal and/or anal wound, such as a recent abdominal scar or anal fissure; meeting any absolute contraindication criteria for the use of abdominal massage therapy, such as an unstable fracture requiring absolute rest and immobilization, pregnancy, or active oncological procedures, and having a cognitive, psychiatric, or neurological condition that, in the clinical judgment of the health care professional, significantly limits the individual’s capacity to provide informed consent or to participate meaningfully in the intervention and data collection procedures.

### Sample and Randomization

Calculations were based on published evidence suggesting that approximately 90% of patients in the control group would remain constipated at 3 months. We hypothesized that this proportion would decrease to 40% in the intervention groups (BI and BIM), corresponding to a 50% absolute difference. To detect this difference with 80% power and a 2-sided significance level of 5%, while allowing for a 10% dropout rate, 15 participants per group are required. With 3 groups, the total sample size will therefore be 45 participants in total. Recruitment will take place across the 2 participating centers (Anoia and Bages), with approximately 7 to 8 participants per group expected to be recruited at each center.

The sample will be selected (1) by means of a medical history review by nursing or medical personnel; if the health professional considers that the patient meets the criteria, they will refer the patient to the research physiotherapist; and (2) indirectly, by putting up posters in *casals cívics* (civic facilities) and pharmacies in the city. Anyone learning about the study this way can make an appointment with their health professional of reference (ie, nursing or medical staff) and discuss their interest in participating in the study. If the health care professional confirms that the patient meets the criteria, they will refer the patient to the research physiotherapist.

The randomization and allocation of patients will be performed by simple randomization using the specialized software RStudio (version 4.2.1; R Foundation for Statistical Computing). The distribution is expected to be homogeneous between groups. Randomization will be conducted by an independent researcher, and allocation will be concealed using sequentially numbered, opaque, sealed envelopes prepared in advance. This procedure ensures allocation concealment and minimizes selection bias.

The chosen sample size is based on feasibility and resource availability and is sufficient to detect large effect sizes. This study aims to assess the feasibility and potential impact of the intervention and to provide effect size estimates to guide future large-scale trials.

The research physiotherapist will inform the participants of the whole process and ask for informed consent, so that they can be randomized into the 3 study groups and their data can be included in the study database for later analysis. Participants will be informed that if the expected results are obtained, they will be offered the same treatment as the intervention group that obtained the best results.

### Intervention

#### BI Group

The behavioral education sessions are structured into two 60-minute workshops delivered in a progressive format. Session 1 focuses on understanding the physiology of defecation, the Rome IV diagnostic criteria, and bowel habits. Session 2 addresses lifestyle-related components, including dietary fiber and hydration guidelines, physical activity recommendations, stress management, optimal defecation posture, and the rational use of laxatives. The sessions are delivered by a multidisciplinary team (nurse, physician, physiotherapist, nutritionist, and psychologist) using multimedia presentations and printed handouts. Participants are encouraged to set personal bowel health goals. Questions and doubts raised during the first session will be discussed and resolved in the second session, ensuring continuity of learning and participant engagement.

The Bristol scale, Rome IV criteria, CVE-20 quality of life questionnaire, and International Physical Activity Questionnaire (IPAQ) [11,29-31] will be explained, and a calendar will be given to the patients to mark when they take any laxatives. Once the information sessions are complete, follow-ups will be scheduled 3 and 6 months later.

#### BIM Group

The BIM group will receive the same intervention as the BI group, and the group will also participate in two 30-minute sessions with the physiotherapist to learn how to perform abdominal massage. In these sessions, the technique will be explained and an explanatory leaflet will be given to the participants so that they can do the massage independently at home.

The abdominal massage protocol follows a standardized clockwise technique that mirrors the path of the colon. The massage begins in the right lower quadrant (ascending colon), progresses to the upper abdomen (transverse colon), and concludes in the left lower quadrant (descending colon). Techniques include gentle effleurage, deep circular kneading, and vibration movements, applied in a slow, consistent rhythm. Each session lasts approximately 30 minutes. Participants are instructed to perform the massage once daily, preferably 20 to 30 minutes after a main meal, in a supine position with knees slightly flexed. The physiotherapist demonstrates the technique and provides a printed visual guide for home practice. The therapist will verify that participants have correctly learned the abdominal massage technique and are able to perform it independently.

To support adherence to the abdominal massage protocol, participants will be provided with a daily self-recording calendar to note each time they perform the massage at home. This calendar will be reviewed at follow-up visits. In addition, weekly SMS reminders will be sent to participants in the BIM group, encouraging them to maintain the daily routine and reinforcing key points of the technique.

Once these sessions are finished, follow-up sessions will be scheduled at 3 and 6 months. Participants will complete the questionnaires again and return the calendar, with marks showing when laxatives were used and massages performed.

#### Control Group

The control group will receive the usual care in their primary care center and will be asked to complete the Bristol scale, Rome IV criteria, CVE-20 quality of life questionnaire, and IPAQ [11,29,30] at the beginning of the study and after 3, 6, and 12 months. They will also be given the calendar to mark when they take laxatives. This calendar will be collected after 3 months.

#### Access to Usual Care

All participants in the study, including those in the BI and BIM groups, will continue to have access to usual care throughout the study period. Usual care refers to the standard clinical management offered by primary care physicians and nurses, which may include dietary advice, physical activity counseling, laxative prescriptions, or follow-up visits, depending on individual needs and clinical judgment. No restrictions are placed on access to routine health care.

### Ethical Considerations

The study was designed in accordance with the principles of the World Medical Association Declaration of Helsinki, as amended in 2013, and applicable regulations. The protocol was approved by the ethics committee of Jordi Gol Primary Care Research Institute (Barcelona, January 22, 2024; code 23/253-P).

The data controller and processor will be the Catalan Institute of Health. The database will be stored for a period of 10 years. The data obtained will not be disclosed to third parties by the research team. International transfers are not provided for. Security mechanisms are sufficient to prevent breaches of confidentiality.

Participants will be informed of the objectives and general aspects of this study and will be asked for informed consent. The informed consent form will specifically include the voluntary nature of participation, security measures, and the confidentiality of the data and its exclusive use for research purposes.

The European Data Protection Regulation 2016/679 of the European Parliament and of the Council of 27 April 2016 on the protection of natural persons with regard to the processing of personal data and the free movement of such data and the Organic Law 3/2018 of 5 December on the Protection of Personal Data and guarantee of digital rights will be complied with at all times.

This protocol has been registered on ClinicalTrials.gov (NCT06359249). The final results will be submitted for publication in peer-reviewed journals and presented at relevant national and international conferences. In addition, findings will be shared with participating primary care centers and regional health authorities to support possible adoption into clinical practice. Participants will be informed of the study results through summary reports distributed via their primary care teams.

## Results

### Progress

Participant enrollment concluded in August 2025, and data collection is ongoing and expected to continue until April 2026. The primary and secondary outcomes will be assessed at 3, 6, and 12 months after the intervention. Results will be analyzed using RStudio and reported in accordance with the Consolidated Standards Of Reporting Trials (CONSORT) guidelines for randomized trials.

### Dissemination Plans

The study’s findings will be disseminated through peer-reviewed publications and presentations at national and international conferences.

### Variables

We will evaluate a set of indicators on constipation and other patient characteristics, as shown in Table 1. Outcomes will be assessed individually and at 5 different time points: before starting the intervention, on the same day of the intervention, 3 months after the intervention, 6 months after the intervention, and 12 months after the intervention. Data will be collected through standardized questionnaires to be completed independently by the participants.

**Table 1.** Study variables.

Study area and instrument		Measurement time point
Constipation		
	Rome IV criteria—degree of constipation	Tpre^a^, T0^b^, T3^c^, T6^d^, T12^e^
	Bristol Stool Form Scale—stool type	T0, T3, T6, T12
Quality of life		
	CVE-20 questionnaire	T0, T3, T6, T12
Use of laxatives		
	Self-recorded calendar of laxative intake	T3, T6, T12
Patient characteristics		
	ECAP^f^—sex, age, previous conditions	Tpre
	IPAQ^g^—physical activity level	T0, T3, T6, T12
Habit change intention		
	Dichotomous question	T0

a Tpre: before the intervention.

b T0: start of the intervention.

c T3: 3 months after the intervention.

d T6: 6 months after the intervention.

e T12: 12 months after the intervention.

f ECAP: Electronic Primary Care Clinical Workstation.

g IPAQ: International Questionnaire of Physical Activity.

### Statistical Analysis

The main variable will be the percentage of patients with constipation 3 and 6 months after the start of treatment for each group, a dichotomous qualitative variable. Prevalence will be estimated and analyzed using the *χ*^2^ test.

The other variables will be reported as percentages and counts for categorical variables and through mean, median, deviation, and quartiles for continuous variables. In order to analyze relationships between the main variable and the sociodemographic or other explanatory variables, the *χ*^2^ test will be used, and the relative risk will be estimated to compare the reduction between the two intervention groups with respect to the control group.

To evaluate the secondary variable, a continuous quantitative variable, ANOVA or the Kruskal-Wallis test will be used to determine differences between the 3 groups. To determine differences between pairs of groups, the Student *t* test or Mann-Whitney test will be used. Analyses will use 95% CIs, and the significance level will be set at 5%.

Due to the nature of the intervention, participant blinding is not feasible. However, to minimize potential bias in outcome assessment, data entry and statistical analysis will be performed by a researcher who is blinded to group allocation. This approach ensures objectivity in the interpretation of results, particularly for subjective outcome measures. All analyses will follow the intention-to-treat principle, including all randomized participants in the groups to which they were originally assigned. Missing data will be handled using multiple imputation under the assumption that data are missing at random. Statistical analysis will be performed with RStudio.

## Discussion

### Principal Anticipated Findings

We anticipate that the structured rehabilitation program combining behavioral reeducation and abdominal massage will lead to a reduction in chronic constipation symptoms and laxative use at 3 and 6 months after the intervention. We also expect improvements in quality of life, particularly among participants receiving both behavioral education and massage therapy. Furthermore, we expect that both intervention groups will show improvements compared to the control group, but that the BIM group will experience greater improvements.

### Comparison to Prior Work

Constipation is often an underestimated condition, and the extent to which people experience hardship from it or its complications is not mentioned in primary care consultations. Reducing constipation will have a positive impact by reducing medicalization, alleviating complications, and decreasing the number of visits to primary care and specialized care. This will result in a better quality of life, reducing personal, physical, and social impacts. In addition, economic expenditures will be reduced due to the lowered use of the aforementioned resources. Therefore, the search for an effective therapy with fewer side effects, both to improve the quality of life of this part of the population and to reduce public health expenditure, should be a topic for research.

It is important to emphasize treating this condition in a logical, step-by-step manner. This includes the establishment of effective and simple conservative measures followed, if necessary, by pharmacological therapy, which is not always necessary. Concerningly, the general population often resorts to pharmacological therapy directly, often without having undergone conservative treatment [1].

The usual treatment for constipation is a reminder of healthy habits, including proper nutrition, hydration, physical activity, and use of laxatives, but these reminders are part of a very short consultation time, often not exceeding 5 to 7 minutes. There is little evidence on other possible conservative techniques, which include lifestyle changes, abdominal massage therapy, anal biofeedback, acupuncture, and dilators [32-39]. This research project guides new multidisciplinary treatments based on a change of habits and a more global approach to health, empowering the patient. In addition, there is the option of abdominal massage, a technique that is less harmful and invasive, less expensive, and self-performed by patient themselves to relieve the symptoms caused by constipation.

### Strengths and Limitations

A key strength of this trial is its pragmatic design, making it well-suited for real-world implementation within the primary care system. The use of validated instruments (the Rome IV criteria, Bristol scale, CVE-20, and IPAQ) ensures reliable outcome measurement. Moreover, the multidisciplinary and participatory nature of the intervention promotes patient empowerment and supports long-term behavior change.

The selection of the counties of l’Anoia and Bages as recruitment areas is based on operational and organizational criteria, as the research team is embedded within the local primary care services, facilitating coordination and participant follow-up. L’Anoia represents a typical urban area in Catalonia (Igualada Urbà Primary Care Center), with demographic and health care characteristics comparable to similar regions, while the inclusion of the Bages region (Navàs Primary Care Center) provides representation of a rural setting. This dual approach ensures feasibility, consistency in intervention delivery, and participant retention, while also improving the external validity of the study by including both urban and rural populations.

One limitation of the study is that the intervention groups receive additional contact with physiotherapists and SMS reminders, while the control group only receives usual care. This greater interaction could introduce attention bias in patient-reported outcomes independently of the abdominal massage itself. Although a sham massage or a time-matched control condition would address this issue, we deliberately adopted a pragmatic design comparing the intervention with standard practice, as this better reflects real-world clinical conditions and enhances external validity.

Another limitation is the absence of a system to verify whether participants in the BIM group correctly perform the abdominal massages at home or whether they perform them at all. This lack of monitoring poses a threat to the internal validity of the study, as the actual exposure to the intervention may vary significantly between individuals. However, during the 2 in-person sessions, the physiotherapist will check that participants are performing the massages correctly. Future protocols should consider incorporating compliance tracking tools or remote follow-up to ensure adherence.

Additionally, the total sample size of 45 participants is relatively small for a 3-arm design with repeated follow-up, which may limit the generalizability of the findings. Moreover, recruitment is restricted to 2 geographic regions (Anoia, representing an urban area, and Bages, representing a rural area), which may affect the representativeness of the study population beyond these contexts.

### Future Directions

Should the findings be favorable, future research should focus on scaling the intervention to other regions and health care systems, increasing the sample size, and assessing long-term outcomes, beyond 6 months. Moreover, future studies could include a sham massage group to control for attention bias and better isolate the effect of abdominal massage.

## Supplementary material

10.2196/72018 Multimedia Appendix 1 Medications for constipation.

## Abbreviations

BI: behavioral intervention
BIM: behavioral intervention and massage
CONSORT: Consolidated Standards Of Reporting Trials
IPAQ: International Questionnaire of Physical Activity
SPIRIT: Standard Protocol Items: Recommendations for Interventional Trials
