# Supplementary material for: A Primary Care Program Based on Behavioral Reeducation and Abdominal Massage for Improving the Symptoms of Chronic Constipation: Protocol for a Randomized Controlled Trial
Source: JMIR Res Protoc. 2025 Dec 4;14:e72018. doi: 10.2196/72018 (PMC12677732; doi:10.2196/72018)
Supplement: Multimedia Appendix 1 [file resprot-v14-e72018-s001.docx]

| **Symptom** | **Medications** | **Observations** |
| --- | --- | --- |
| Constipation ^1-3^ | Antacids with aluminum  H2 antihistamines  IBP  Sucralfate  Laxatives (chronic use)  Calcium supplements  Iron supplements  Amiodarone  Ranolazine  Diuretics  Beta-blockers  Calcium channel blockers  Cholestyramine, colestipol  Oral contraceptives  Urinary antispasmodics  NSAIDs  Bisphosphonates |  |
|  | Opioids | Its prescription should be accompanied by preventive measures, including laxatives. |
|  | Antipsychotics  Anticholinergic antiparkinsonian drugs  Lithium  Tricyclic antidepressants, duloxetine , venlafaxine H1 antihistamines |  |

Appendix – Medication and Constipation

**Bibliographic references**

1. Jain V, Pitchumoni CS. Gastrointestinal side effects of prescription medications in the older adults. J Clin Gastroenterol 2009;43(2):103-10.
2. Triantafyllou K, Vlachogiannakos J, Ladas SD. Gastrointestinal and liver side effects of drugs in elderly patients. Best Pract Res Clin Gastroenterol 2010;24(2): 203-15.
3. Lat I, Foster DR, Erstad B. Drug-induced acute liver failure and gastrointestinal complications. Crit Care Med 2010;38(6 Suppl ):175-87.
